# Sexual abuse and physical neglect in childhood are associated with affective theory of mind in adults with schizophrenia

**DOI:** 10.1016/j.scog.2020.100189

**Published:** 2020-10-23

**Authors:** Anja Vaskinn, Ingrid Melle, Monica Aas, Akiah Ottesen Berg

**Affiliations:** a Norwegian Centre for Mental Disorders Research, Oslo University Hospital, Oslo, Norway; b Institute of Clinical Medicine, University of Oslo, Oslo, Norway

**Keywords:** Social cognition, Psychosis, Mentalizing, Maltreatment, Adversity, Mind-reading

## Abstract

Whereas childhood trauma is associated with reduced nonsocial cognition in schizophrenia, research on the relationship between childhood trauma and social cognition is limited and mixed. The aim of this study was to examine the association between childhood trauma and theory of mind (ToM) in persons with schizophrenia (n = 68) compared to healthy control participants (n = 70). Childhood trauma was assessed with the Childhood Trauma Questionnaire (CTQ), providing information on physical abuse, emotional abuse, sexual abuse, physical neglect and emotional neglect. ToM was indexed by the Movie for the Assessment of Social Cognition (MASC), which yields scores for total, cognitive and affective ToM, and for three error types (overmentalizing, undermentalizing, no mentalizing). Persons with schizophrenia had elevated rates of childhood trauma and lower ToM scores than healthy controls. In the schizophrenia group, associations between sexual abuse and affective ToM was statistically significant. In regression analyses, physical neglect was found to be the strongest predictor of affective ToM. In healthy controls, childhood trauma was not associated with ToM. Follow-up analyses comparing individuals with/without clinically significant childhood trauma, confirmed the findings for the schizophrenia group. No causal inferences can be made in this cross-sectional study, but the results suggest an illness-specific association between both sexual abuse and physical neglect in childhood, and adult affective ToM in individuals with schizophrenia.

## 1 Introduction

Childhood trauma is prevalent in individuals with schizophrenia (Varese et al., 2012), increases the risk of psychosis (Bendall et al., 2008; Varese et al., 2012) and is among stress-related environmental risk factors implicated in the development of psychosis (Misiak et al., 2017). Interpersonal childhood trauma, i.e. physical abuse, emotional abuse, sexual abuse, physical neglect and/or emotional neglect, are not only potent predictors of psychotic disorder (Schäfer and Fischer, 2011) or psychotic experiences (van Nierop et al., 2014), but also associated with severity of positive symptoms (Bailey et al., 2018) and slower improvement rates (Aas et al., 2016) in persons with a psychotic disorder.

Individuals with schizophrenia also have social cognitive impairments (Savla et al., 2013), i.e. deficits in their ability to “perceive, interpret and generate responses to the intentions, dispositions and behavior of others” (Green et al., 2008). Meta-analytic evidence shows that schizophrenia is characterized by reduced ability to decode the emotional expressions of others (emotion perception), to identify social roles, rules and contexts (social perception), and to infer the mental state of others (mentalizing/theory of mind (ToM)) (Savla et al., 2013). These social cognitive impairments, especially ToM, are strong predictors of outcome in schizophrenia (Fett et al., 2011).

Childhood trauma and social and nonsocial cognitive impairments are not only common features of schizophrenia, but may also be linked. There are several possible explanations of why. One is that reduced cognition may result from the negative effect of childhood adversity on neuronal development, shown to disrupt the hypo-thalamic-pituitary axis (HPA) (Agorastos et al., 2018). This is at the heart of the traumagenic model, which states that early trauma leads to abnormal neurodevelopment that again underlies the heightened responsivity to stress, such as overactivity of the HPA axis (Read et al., 2001). There are also psychological explanations of why childhood trauma may have a negative impact on adult cognition. As for ToM, its development rests on a combination of innate developmental processes and external learning experiences that depend on the environment surrounding the child. ToM therefore probably has both genetic (Bora et al., 2009) and environmental (Stanzione and Schick, 2014) underpinnings. Heyes and Frith (2014) proposed a distinction between early innate neurocognitive mechanisms that enable nonliterate infants to predict behavior, that is, implicit mindreading, and a culturally inherited skill of explicit mindreading, transferred to the next generation through verbal instruction. A child who experiences childhood trauma may lack access to adults who are able or willing to provide learning experiences of how to think about the mental state of others, i.e. adults who talk less about feelings and beliefs. A meta-analysis found that maltreatment has a negative effect on children's social understanding, including their ToM (Luke and Banerjee, 2013). There appears to be a stronger relationship with processing of social information concerning emotions than thoughts (Luke and Banerjee, 2013; Benarous et al., 2015).

Other research indicates that childhood trauma can have a negative effect on cognition also in adults, both healthy individuals and persons with psychosis (Misiak et al., 2017). Two recent systematic reviews of the relevant literature concluded that childhood trauma is associated with reduced cognitive function in psychosis (Vargas et al., 2019; Dauvermann and Donohoe, 2019). Although three social cognitive studies were included in one of the reviews (Dauvermann and Donohoe, 2019), they were not interpreted because they were few with inconsistent results. However, a systematic literature review across major psychiatric disorders concluded that early negative social experiences, including childhood trauma, seems to be associated with poorer social cognition (Rokita et al., 2018).

The emerging literature on childhood trauma and adult social cognition in psychosis suggests that neglect may be of particular importance. Three studies investigated the association using the MATRICS Consensus Cognitive Battery (MCCB) (Nuechterlein et al., 2008) where social cognition is represented by a measure of emotional processing (Mayer et al., 2002). In a Spanish study of participants with early psychosis (< 3 years of illness duration) childhood trauma (total score on the commonly used Childhood Trauma Questionnaire: CTQ) contributed significantly to social cognition, but not to the other cognitive domains in the MCCB (Garcia et al., 2016). This was shown in multiple regression analysis using the total CTQ score, but was also reflected in strong and significant correlation coefficients between the social cognitive test and CTQ, more specifically the total score and the two neglect subscales of the CTQ (emotional neglect, physical neglect) (Garcia et al., 2016). In a clever study, Schalinski et al. (2018) were able to examine how childhood adverse events at different developmental stages (ages) related to adult social and nonsocial cognition (MCCB domains) in participants within the psychosis spectrum. The results of this German study suggest that the timing of adverse childhood events matter. Early abuse (at age 3) was associated with impairments in attention, learning and working memory, whereas neglect in pre-adolescence (age 11–12) was related to reduced social cognition (Schalinski et al., 2018). The findings were interpreted within the traumagenic neurodevelopmental model (Read et al., 2014). The impact of early abuse on cognitive functions like attention and memory, and of later neglect on higher social cognitive functions, aligns with sensitive periods for neurodevelopment. The hippocampus, which develops early, is of relevance for attention and memory, whereas prefrontal areas, central to higher order processes including social cognition, mature later (Bick and Nelson, 2016). In a study from South Africa, Kilian et al. (2018) also found an association between neglect (CTQ physical and emotional neglect subscales, collapsed) and social cognition in persons with first-episode schizophrenia spectrum disorder. This association was not present for abuse nor illness-specific as it was found also in healthy control participants.

ToM has been examined in three studies. Palmier-Claus et al. (2016) examined if several clinical and cognitive factors, including ToM, served as mediators between childhood trauma and social functioning. ToM was assessed with the Hinting Task (Corcoran et al., 1995) and the Reading the Mind in the Eyes Test (Baron-Cohen et al., 2001). Childhood trauma, indexed by the total CTQ score, did not predict ToM, and was therefore not considered to be a mediator (Palmier-Claus et al., 2016). This study from the United Kingdom used a mixed sample consisting of persons with either established schizophrenia, first episode psychosis or with ultra-high risk for developing psychosis. One study from Northern Ireland examined if political violence and childhood trauma were associated with ToM in schizophrenia (Kincaid et al., 2018). This study also used the Hinting Task, and this time childhood trauma, more specifically the emotional neglect subscale of the CTQ, did indeed predict ToM. Finally, in their study from Ireland, Rokita et al. (2020) found physical neglect to be associated with emotion recognition, but not with ToM assessed with the Reading the Mind in the Eyes test. In summary, the existing studies indicate that childhood neglect may be more important than childhood abuse to adult social cognition in psychosis (Kincaid et al., 2018; Schalinski et al., 2018; Kilian et al., 2018; Rokita et al., 2020).

The social cognitive measures used in the above-mentioned studies have all been criticized, for various reasons. The Reading the Mind in the Eyes Test may be a measure of emotion recognition and not of ToM (Oakley et al., 2016), the Hinting Task can produce ceiling effects (Davidson et al., 2018; Frøyhaug et al., 2019), and the MCCB subtest may be a poor operationalization of social cognition in younger participants (Holmén et al., 2010). Further, none of them has convincing ecological validity, i.e. the test stimuli are not very similar to the shifting, dynamic social cognitive stimuli of the real world. The Movie for the Assessment of Social Cognition (MASC) test (Dziobek et al., 2006) may be a better alternative. The MASC is a valid and reliable measure (Dziobek et al., 2006), able to identify ToM impairments in schizophrenia (Montag et al., 2011; Andreou et al., 2015; Vaskinn et al., 2015). It consists of a video film where four characters, two males and two females, interact in common social situations. Such test stimuli increase the ecological validity. The MASC test also has other advantages. It provides information on cognitive and affective ToM (knowledge of another's thoughts and emotions, respectively), as well as on the participants' mentalizing style (over- versus undermentalizing). Thus, compared to other tests, the MASC test enables a more thorough assessment of ToM.

Although ToM performance in individuals with schizophrenia, assessed with the MASC test, is associated with clinical symptoms (Fretland et al., 2015), low-level emotion perception (Vaskinn et al., 2018) and nonsocial cognition (Sjølie et al., 2020), neither of these characteristics can fully explain the ToM impairments in schizophrenia. It is therefore likely that ToM is determined also by other factors, and childhood trauma may be among these.

In the current study, we examine the association between childhood trauma and ToM in adults with schizophrenia, compared to healthy control participants. It is a follow-up study using a sample partly overlapping with those of previous publications from our research group (Fretland et al., 2015; Vaskinn et al., 2018; Sjølie et al., 2020). For individuals with schizophrenia, we hypothesize significant associations between childhood trauma, especially neglect, and ToM. We make no predictions for error types, but anticipate stronger associations with affective than cognitive ToM. We hypothesize negligible associations in healthy control participants, given their expected intact social cognition and lower rates of childhood trauma.

## 2 Methods

### 2.1 Participants

Sixty-eight individuals with schizophrenia (n = 54) or schizoaffective disorder (n = 14) were included, along with 70 healthy control participants, from 2010 to 2017. All participated in the larger Thematically Organized Psychosis (TOP) study at Oslo University Hospital in Oslo, Norway. All participants signed informed consent after having received oral and written information about the study. The study was approved by the regional committee for medical research ethics and was completed in accordance with the Helsinki Declaration. Inclusion criteria were age between 18 and 55 years, and Norwegian mother tongue or all compulsory education received in Norway. Exclusion criteria were head injury causing hospitalization, neurological disease, and IQ < 70 as assessed with the 2-subtest Wechsler Abbreviated Scale of Intelligence (WASI) (Wechsler, 2007). Participants with schizophrenia were recruited from hospitals in the Oslo area (11 inpatients/57 outpatients), whereas healthy control participants were randomly selected from official population registries of the same geographical areas and invited by letter to participate. Healthy controls were only included if mental, neurological or somatic disorder was disconfirmed through screening using the Primary Care Evaluation of Mental Disorders interview (PRIME-MD) (Spitzer et al., 1994). Demographic information is presented in Table 1.

**Table 1:** Demographics and clinical characteristics in individuals with schizophrenia (n = 68) and in healthy control participants (n = 70).

	Schizophrenia (n = 68)	Healthy control participants (n = 70)	Statistic	
	Mean (SD)	Mean (SD)	Value	Sig
Demographics				
Age	29.4 (8.1)	29.4 (7.7)	t = −0.02	p = 0.696
Sex (males/females)	43/25	42/28	X^2^ = 0.15	p = 0.696
Education (years)	12.3 (2.5)	14.2 (2.1)	t = −4.87	p < 0.001
WASI IQ	100.9 (13.0)	110.9 (10.8)	t = −4.92	p < 0.001
Clinical features				
PANSS positive subscale (range 7–49)	14.0 (4.8)	–	–	–
PANSS negative subscale (range 7–49)	15.1 (5.3)	–	–	–
CDSS^a^ (range 0–13)	4.3 (3.5)	–	–	–
Illness duration^b^	7.0 (7.2)	–	–	–
Inpatients/outpatients	11/57	–	–	–
Medication^c^	1.45	–	–	–

WASI = Wechsler Abbreviated Scale of Intelligence, PANSS = Positive and Negative Syndrome Scale, CDSS = Calgary Depression Scale for Schizophrenia.

a n = 61 due to missing data.

b n = 66 due to missing data.

c Amount of defined daily dose of antipsychotic treatment, n = 59.

### 2.2 Materials

#### 2.2.1 Clinical instruments

Diagnoses were based on the Diagnostic and Statistical Manual for Mental Disorders (APA, 2000) using the Structured Clinical Interview for DSM-IV (SCID-I) (First et al., 1996). Psychopathology was measured with the Positive and Negative Syndrome Scale (PANSS) (Kay et al., 1987) and Calgary Depression Scale for Schizophrenia (Addington et al., 1993). Clinical information is presented in Table 1.

#### 2.2.2 Assessment of childhood trauma

Childhood trauma was measured with the short screening version of the Childhood Trauma Questionnaire (CTQ; Bernstein et al., 2003). This self-report measure assesses five types of traumatic experiences (emotional, physical and sexual abuse, emotional and physical neglect). CTQ contains 28 statements that are rated on a 5-point scale from 1 (never true) to 5 (very often true). Its five subscales correspond to the five trauma types and all have five items.

In addition to using the continuous scores of each subscale, we dichotomized the CTQ subscale scores using the severity criteria recommended by Walker et al. (1999). These cut-off criteria stem from a comparison of self-reported CTQ scores with expert ratings of detailed clinical interviews concerning whether clinically significant childhood trauma was present (Glaesmer, 2016). See Table 2 for the cut-off criteria for the five CTQ subscales. The last three CTQ items belong to the response bias subscale, the minimization and denial (MD) scale. Unlike the items of the five abuse/neglect subscales, the MD items are dichotomized: scores of 5 (very often true) are coded as 1 and scores 1 through 4 are coded as 0 (Bernstein and Fink, 1998). Individuals who score ≥ 1 for at least one MD item are considered “MD positive” (MD+); indicative of an overly positive, idyllic representation of childhood experiences (MacDonald et al., 2016).

**Table 2:** Childhood trauma severity and theory of mind performance in individuals with schizophrenia (n = 68) compared to healthy control participants (n = 70).

	Schizophrenia (n = 68)	Healthy control participants (n = 70)	Statistic	
			Value	Sig
Childhood trauma	Median (range) n (%) > cut-off	Median (range) n (%) > cut-off		
CTQ total score	37 (25–86)	27 (25–53)	U = 801.0	p < 0.001
(range 25–125)				
	–	–	–	–
CTQ physical abuse	5 (5–17)	5 (5–11)	U = 1717.0	p < 0.001
(range 5–25)				
n (%) > cutoff ≥8	10 (14.7%)	2 (2.9%)	X^2^ = 5.97	p = 0.015
CTQ sexual abuse	5 (5–25)	5 (5–7)	U = 1983.0	p = 0.005
(range 5–25)				
n (%) > cutoff ≥8	9 (13.6%)	0 (0%)	X^2^ = 10.22	p = 0.001
CTQ emotional abuse	9 (5–25)	5 (5–18)	U = 1027.0	p < 0.001
(range 5–25)				
n (%) > cutoff ≥10	26 (38.8%)	6 (8.6%)	X^2^ = 17.48	p < 0.001
CTQ emotional neglect	10 (5–22)	6 (5–15)	U = 984.0	p < 0.001
(range 5–25)				
n (%) > cutoff ≥15	19 (28.4%)	1 (1.5%)	X^2^ = 19.62	p < 0.001
CTQ physical neglect	6 (5–13)	5 (5–13)	U = 1623.5	p < 0.001
(range 5–25)				
n (%) > cutoff ≥8	21 (30.9%)	9 (12.9%)	X^2^ = 6.59	p = 0.010
CTQ minimization/denial	0 (0–3)	0 (0–3)	U = 1775.0	p = 0.016
(range 0–3)				
MD+ n (%) ≥ 1	19 (27.9%)	31 (47.0%)	X^2^ = 5.19	p = 0.023
				
	Mean (SD)	Mean (SD)		
	0.47 (0.86)	0.89 (1.11)	t = −2.47	p = 0.015

Theory of mind	Mean (SD)	Mean (SD)		
MASCtot (range 0–45)	29.2 (7.0)	35.2 (4.1)	t = −6.16	p < 0.001
MASCcog correct (range 0–26)	17.2 (4.6)	21.3 (2.7)	t = −6.38	p < 0.001
MASCaff correct (range 0–18)	11.7 (2.9)	13.6 (1.9)	t = −4.41	p < 0.001
MASCexc errors (range 0–45)	5.5 (3.9)	4.4 (2.8)	t = 1.95	p = 0.053
MASCless errors (range 0–45)	6.6 (3.2)	3.9 (2.0)	t = 6.00	p < 0.001
MASCno errors (range 0–45)	3.7 (2.4)	1.5 (1.4)	t = 6.56	p < 0.001

CTQ = Childhood Trauma Questionnaire, MD+ = MD positive, MASC = Movie for the Assessment of Social Cognition, MASCtot = MASC total score, MASCcog = MASC cognitive ToM; MASCaff = MASC affective ToM, MASCexc = MASC overmentalizing errors, MASCless = MASC undermentalizing errors, MASCno = MASC no mentalizing errors. U = Mann-Whitney *U* test, X^2^ = Chi-square test, t = Independent samples *t*-test. Missing data in the schizophrenia group: 2 for sexual abuse, 1 for emotional abuse, 1 for emotional neglect, 4 for total trauma score. Missing data among healthy controls: 1 for physical abuse, 1 for emotional neglect, 2 for total trauma score, 4 for minimization/denial.

#### 2.2.3 Measure of theory of mind (ToM)

ToM was assessed with the Norwegian version of the MASC test (Fretland et al., 2015). The four characters in the MASC film are shown in different situations, including during a dinner party. The movie, administered on a computer using the PowerPoint software, is paused 45 times for questions to be asked about a given character's thoughts, emotions, or intentions. Responses are given orally and recorded by the test administrator. Total administration time for the MASC test is 30–40 min. As mentioned in the Introduction, the MASC test provides several ToM scores. In addition to the overall score (MASCtot), the questions can be allocated into affective (MASCaff) or cognitive (MASCcog) ToM. Affective ToM concerns an empathic understanding of the other's emotional state (knowledge about *emotions*), whereas cognitive ToM entails a cognitive understanding of what the other knows or thinks (knowledge about *beliefs*) (Shamay-Tsoory et al., 2007). We used the allocation of items to MASCcog and MASCaff as described previously (Vaskinn et al., 2018). Further, as the questions are answered by ticking one of four response options, one of which is correct, the test provides information about the types of errors, that is, the mentalizing style, of a respondent. There are three types of errors. Overmentalizing errors (MASCexc) are exaggerated, overly interpretive responses. Undermentalizing errors (MASCless) are responses where attributions are made to internal mental states, but they are underinterpretive. Finally, in the case of errors of the No mentalizing (MASCno) type, the participant does not make any attributions to the mental state of the character in question. See Appendix A for examples.

### 2.3 Statistical analyses

Tests of normality indicated that all CTQ measures were non-normally distributed (Kolmogorov-Smirnov ps < 0.001 except CTQ emotional abuse p = 0.009). This was confirmed by visual inspection of histograms that were substantially skewed. Tests of normality suggested that some MASC variables were non-normally distributed, but histograms were largely in line with a normal distribution. Therefore, non-parametric tests were used for analyses involving CTQ variables, whereas parametric tests were used for analyses of MASC only variables. Statistical analyses were undertaken with The Statistical Package for the Social Sciences (IBM SPSS Statistics for Windows, Version 26.0, IBM Corp, Armonk, NY).

To establish the relevance of previous research for the current sample, group differences in CTQ scores and in MASC performance, were examined with Mann-Whitney *U* tests and independent samples *t*-tests, respectively.

Our research aim regarding associations between childhood trauma and ToM was investigated in two steps. In the first step, Spearman's rho correlations between CTQ and MASC were calculated in the schizophrenia and healthy control groups, respectively (including follow-up analyses excluding MD+ participants). The significance level was adjusted for multiple comparisons. The new p-level (two-sided) was 0.01 (five correlations for each MASC score: p-value 0.05/5 CTQ subscales = 0.01). In the second step, variables with a statistically significant association in the first step were entered in regression analyses; MASC score(s) as the dependent variable and CTQ subscale(s) as independent variable(s). Models were adjusted for age and IQ (but not sex as we have previously shown that males and females do not differ in MASC performance: Fretland et al., 2015). Assumptions for linear regression were checked and found satisfactory. Visual inspection of normal probability plot of standardized residuals suggested normality. Homoscedasticity was confirmed by the Breusch-Pagan test. Together this confirmed linearity. There was no multicollinearity (all inter-correlations for age, IQ, and CTQ subscales <0.70, all VIFs <2.5).

As follow-up analyses, group comparisons of MASC performance between individuals with and without clinically significant childhood trauma (Walker et al., 1999; Glaesmer, 2016) on CTQ subscales found to be significantly associated with MASC in the preceding analyses were undertaken. This was also done separately in the schizophrenia and healthy control groups. Independent samples *t*-tests with Cohen's *d* (using the pooled standard deviation) as a measure of effect size were applied.

## 3 Results

### 3.1 Group differences in childhood trauma and ToM performance

Group differences in traumatic childhood experiences and in ToM are presented in Table 2. Mann-Whitney U analyses showed that the schizophrenia group had substantially higher CTQ scores than healthy controls. This was evident for the total score as well as for the CTQ subscales. Rates of clinically significant childhood trauma were also higher in the schizophrenia group. Group differences were highly statistically significant (all p-values ≤0.01). Significant group differences were also present for MD, but numbers indicated that MD+ status was more common in healthy controls than in persons with schizophrenia. Independent samples *t*-tests yielded significant group differences for all MASC variables, except for MASCexc, with the schizophrenia group performing worse.

### 3.2 The relationship between childhood trauma and ToM performance

The Spearman rho's correlation coefficients from the first step of our analyses of associations between childhood trauma and ToM are shown in Table 3 (schizophrenia group) and Table 4 (healthy controls).

**Table 3:** Correlation coefficients (Spearman's *rho*) between theory of mind and childhood trauma in participants with schizophrenia (n = 68).

	MASCtot -correct	MASCaff -correct	MASCcog -correct	MASCexc -errors	MASCless -errors	MASCno -errors
CTQ total^a^	−0.034 p = 0.791	−0.151 p = 0.235	0.031 p = 0.805	0.022 p = 0.861	0.032 p = 0.799	0.050 p = 0.695
CTQ physical abuse	−0.179 p = 0.145	−0.232 p = 0.057	−0.155 p = 0.207	0.132 p = 0.283	0.107 p = 0.383	0.128 p = 0.299
CTQ sexual abuse^b^	−0.218 p = 0.079	**−0.321**⁎ **p =** **0.009**	−0.159 p = 0.202	0.144 p = 0.248	0.200 p = 0.107	0.178 p = 0.152
CTQ emotional abuse^c^	−0.002 p = 0.985	−0.080 p = 0.519	0.023 p = 0.854	0.087 p = 0.484	−0.085 p = 0.494	−0.075 p = 0.546
CTQ emotional neglect^d^	0.021 p = 0.865	−0.069 p = 0.579	0.086 p = 0.489	−0.076 p = 0.539	−0.003 p = 0.984	0.062 p = 0.618
CTQ physical neglect	−0.214 p = 0.080	−0.287 p = 0.017	−0.157 p = 0.201	0.076 p = 0.538	0.247 p = 0.043	0.216 p = 0.077

CTQ = Childhood Trauma Questionnaire, MASC = Movie for the Assessment of Social Cognition, MASCtot = MASC total score, MASCcog = MASC cognitive ToM; MASCaff = MASC affective ToM, MASCexc = MASC overmentalizing errors, MASCless = MASC undermentalizing errors, MASCno = MASC no mentalizing errors. Missing data: ^a^ n = 4, ^b^ n = 2, ^c^ n = 1, ^d^ n = 1.

⁎ Significant at the adjusted p-level (**p < 0.01**).

**Table 4:** Correlation coefficients (Spearman's *rho*) between theory of mind and childhood trauma in healthy control participants (n = 70).

	MASCtot -correct	MASCaff -correct	MASCcog -correct	MASCexc -errors	MASCless -errors	MASCno -errors
CTQ total^a^	−0.072 p = 0.562	−0.123 p = 0.316	−0.002 p = 0.989	0.100 p = 0.418	0.064 p = 0.604	−0.018 p = 0.886
CTQ physical abuse^b^	0.118 p = 0.334	−0.014 p = 0.908	0.183 p = 0.133	−0.120 p = 0.327	0.096 p = 0.431	−0.254 p = 0.035
CTQ sexual abuse	0.088 p = 0.471	0.030 p = 0.804	0.127 p = 0.296	−0.086 p = 0.480	0.037 p = 0.763	−0.218 p = 0.069
CTQ emotional abuse	0.041 p = 0.739	−0.034 p = 0.782	0.088 p = 0.469	0.004 p = 0.976	0.082 p = 0.499	−0.205 p = 0.089
CTQ emotional neglect^c^	−0.084 p = 0.495	−0.108 p = 0.377	−0.021 p = 0.866	0.129 p = 0.291	0.011 p = 0.928	0.028 p = 0.820
CTQ physical neglect	0.000 p = 0.999	−0.068 p = 0.575	0.054 p = 0.658	−0.112 p = 0.356	0.056 p = 0.643	0.067 p = 0.580

CTQ = Childhood Trauma Questionnaire, MASC = Movie for the Assessment of Social Cognition, MASCtot = MASC total score, MASCcog = MASC cognitive ToM; MASCaff = MASC affective ToM, MASCexc = MASC overmentalizing errors, MASCless = MASC undermentalizing errors, MASCno = MASC no mentalizing errors. Missing data: ^a^ n = 2, ^b^ n = 1, ^c^ n = 1.

In the schizophrenia group, only the correlation coefficient between sexual abuse and MASCaff (Spearman's rho = −0.321, p = 0.009) was statistically significant at the adjusted p-level. The bivariate association between physical neglect and MASCaff was medium-sized, but did not reach the corrected p-level for significance (Spearman's rho = −0.287, p = 0.017). All correlations indicated that higher level of childhood trauma was related to worse ToM. No significant associations were present in the healthy control group.

Follow-up analyses in the schizophrenia sample excluding MD+ participants, confirmed that the strongest associations were present for correlation coefficients between MASCaff and sexual abuse (Spearman's rho = −0.373, p = 0.009) and physical neglect (Spearman's rho = −0.395, p = 0.005), respectively. Similar confirmation resulted from follow-up analyses among healthy controls excluding MD+ participants: none of the correlation coefficients were significant.

In the second step of analyses of associations between childhood trauma and ToM, a standard linear regression with sexual abuse entered as an independent variable (along with age and IQ) and MASCaff as the dependent variable was conducted for participants with schizophrenia. The model was significant (R^2^ = 0.204, Adjusted R^2^ = 0.165, F_(3,62)_ = 5.29, p = 0.003) with a nominally significant contribution from sexual abuse (β = −0.21, *t* = −1.85, p = 0.069). Given its trend-level significant association with MASCaff (Spearman's rho = 0.29, p = 0.017) and the existing findings of its potential as a predictor of social cognition, physical neglect was entered as a second independent variable, together with sexual abuse, in a post-hoc standard linear regression (MASCaff as dependent variable). This model was also significant (R^2^ = 0.282, Adjusted R^2^ = 0.235, F_(4,61)_ = 5.99, p < 0.001), but with physical neglect in the model sexual abuse was not a unique contributor to MASCaff (β = −0.04, t = −0.32, p = 0.752). Physical neglect contributed significantly to MASCaff (β = −0.33, t = −2.58, p = 0.012).

### 3.3 Comparisons between individuals with and without clinically significant childhood trauma

Follow-up analyses comparing the MASC scores of schizophrenia participants with (n = 9) and without (n = 57) clinically significant childhood sexual abuse is presented in Table 5. A preliminary, medium-sized, non-significant group difference appeared for MASCaff (Cohen's *d* = 0.61). The MASC scores of schizophrenia participants with (n = 21) and without (n = 47) clinically significant childhood physical neglect can be seen in Table 6. The groups differed significantly for MASCless, with a medium effect size (Cohen's *d* = 0.59). A medium-sized group difference was also present for MASCaff (Cohen's *d* = 0.49), but this was non-significant.

**Table 5:** Theory of mind in schizophrenia participants with and without childhood sexual abuse.

	With childhood sexual abuse n = 9	Without childhood sexual abuse n = 57	Statistic t-value	Statistic p-value	Statistic Cohen's d
MASCtot correct (range 0–45)	26.8 (6.3)	29.7 (6.7)	1.25	p = 0.216	0.45
MASCcog correct (range 0–26)	15.9 (4.7)	17.5 (4.4)	1.03	p = 0.306	0.34
MASCaff correct (range 0–18)	10.6 (1.8)	12.0 (2.8)	1.44	p = 0.154	0.61
MASCexc errors (range 0–45)	6.6 (2.6)	5.2 (3.6)	1.12	p = 0.268	0.45
MASCless errors (range 0–45)	7.4 (4.2)	6.5 (3.0)	0.86	p = 0.392	0.25
MASCno errors (range 0–45)	4.2 (2.5)	3.7 (2.4)	0.66	p = 0.511	0.20

MASC = Movie for the Assessment of Social Cognition, MASCtot = MASC total score, MASCcog = MASC cognitive ToM; MASCaff = MASC affective ToM, MASCexc = MASC overmentalizing errors, MASCless = MASC undermentalizing errors, MASCno = MASC no mentalizing errors.

**Table 6:** Theory of mind in schizophrenia participants with and without childhood physical neglect.

	With childhood physical neglect n = 21	Without childhood physical neglect n = 47	Statistic t-value	Statistic p-value	Statistic Cohen's d
MASCtot correct (range 0–45)	27.0 (7.1)	30.2 (6.8)	1.76	p = 0.084	0.46
MASCcog correct (range 0–26)	16.0 (4.8)	17.8 (4.4)	1.49	p = 0.142	0.39
MASCaff correct (range 0–18)	10.8 (2.8)	12.2 (2.9)	1.85	p = 0.069	0.49
MASCexc errors (range 0–45)	5.9 (3.3)	5.3 (4.2)	0.55	p = 0.585	0.16
MASCless errors (range 0–45)	7.9 (3.8)	6.0 (2.7)	2.42	p = 0.018	0.59
MASCno errors (range 0–45)	4.2 (2.4)	3.5 (2.4)	1.05	p = 0.299	0.29

MASC = Movie for the Assessment of Social Cognition, MASCtot = MASC total score, MASCcog = MASC cognitive ToM; MASCaff = MASC affective ToM, MASCexc = MASC overmentalizing errors, MASCless = MASC undermentalizing errors, MASCno = MASC no mentalizing errors.

## 4 Discussion

This study examined the association between childhood trauma and ToM in individuals with schizophrenia and in healthy control participants. Findings confirmed that individuals with schizophrenia have experienced more childhood trauma than healthy people (Varese et al., 2012; Larsson et al., 2013; Church et al., 2017), and that they have impaired ToM (Savla et al., 2013; Vaskinn et al., 2018). More importantly, we found that childhood trauma of physical neglect and sexual abuse were associated with reduced affective ToM in those with schizophrenia.

Our study aligns with previous studies (Kilian et al., 2018; Schalinski et al., 2018; Rokita et al., 2020) in showing that neglect is of importance to adult social cognition in schizophrenia. Physical neglect was a significant unique predictor of affective ToM and had a non-significant, but not minor association with undermentalizing errors (Spearman's rho = 0.247). Our design cannot confirm causal relationships, but this at least suggests that physical neglect may influence ToM by making the person unable to fully appreciate the emotional mental state of the other. The importance of undermentalizing is supported by the largest effect size in follow-up comparisons of schizophrenia participants with and without a history of childhood physical neglect.

Previous studies did not report a significant association of social cognition with sexual abuse in schizophrenia, perhaps due to the instruments used. Earlier studies used tests that do not provide detailed information beyond a total, overall score. In accordance with those studies, our total MASC score was not significantly associated with sexual abuse. However, when differentiating between types of ToM, a significant association appeared between affective ToM and sexual abuse. It therefore seems that in the case of schizophrenia, a differentiation of ToM is needed to reveal otherwise undetected features associated with childhood maltreatment. For other psychiatric disorders, this may be different. For example, one study identified an association between ToM, indexed by the total MASC score, and sexual assault in borderline personality disorder (Preißler et al., 2010). Sexual abuse does not appear to exert its effect on ToM through a specific mentalizing style, reflected in similarly sized correlation coefficients for the error types as well as similar (small) effect sizes in follow-up group comparisons of error types between schizophrenia participants with or without a history of sexual abuse. However, the effect of sexual abuse on affective ToM diminished when considering the effect of physical abuse. This implies that a feature shared with physical neglect can explain its impact, and that its small effect may be another reason why few studies have found an association with social cognition in schizophrenia.

Interestingly, childhood maltreatment was more strongly associated with affective ToM than with cognitive ToM. One explanation for this may be that the interpersonal betrayal involved in childhood trauma may cause confusion when making inferences about the emotional state of others. The lack of interpersonal trust stemming from experiencing childhood maltreatment may be less consequential for the monitoring of others for their (malign) intentions or thought content (cognitive ToM). This is supported by reviews of maltreated children that show strong evidence for deficits in emotion understanding and emotion recognition (Luke and Banerjee, 2013), but mixed results for cognitive ToM (Luke and Banerjee, 2013; Benarous et al., 2015). The importance of early interpersonal experiences for later emotional processing is also reflected in studies showing that anxious attachment is related to reduced accuracy in facial emotion perception (Fraley et al., 2006). In addition, insecure attachment in schizophrenia may be more related to affective (Pos et al., 2015) than cognitive ToM (Korver-Nieberg et al., 2013; Pos et al., 2015) which may not be surprising given that attachment involves regulation of affects.

For schizophrenia, a few studies have reported associations between childhood trauma and altered brain function in regions relevant for social cognition (the amygdala, the precuneus/posterior cingulate cortex and temporo-parietal junction) during social cognitive tasks (Cancel et al., 2017; Quidé et al., 2017). Overall, childhood trauma in schizophrenia is reliably related to decreased total cerebral grey matter and alterations in white matter integrity (Cancel et al., 2019). But alterations in brain structure and function, although consistently linked to childhood trauma, depend on the type and timing of maltreatment (Teicher and Samson, 2016). Our study which linked specific types of childhood trauma to specific types of ToM aligns with this. As neglect and abuse have distinct effects on neuronal development (McLaughlin et al., 2014), different effects on social cognition may follow. For instance, reduced amygdala volume has been reported for neglect, increased amygdala volume for abuse (Teicher and Samson, 2016). Neglect means understimulation, of both neural structures and of opportunities to think and talk in mental state terms, perhaps resulting in a mentalizing style characterized by “too little” ToM as our study suggests. Since a complex interplay of biopsychosocial processes is present when a young brain develops in a hostile interpersonal context, childhood trauma may have diverse consequences for adult cognition.

The relationship between childhood trauma and ToM was different in our healthy comparison sample, with no significant associations. This suggests that ToM in healthy individuals, an intact social cognitive function, is not determined by childhood trauma.

The tendency to present childhood experiences as idyllic (MD+) was more prevalent in healthy controls than in individuals with schizophrenia, in line with international literature (MacDonald et al., 2016) and a larger previous study that included some of the current participants (Church et al., 2017). The MD+ rates in our sample (healthy sample: 47%, schizophrenia sample: 28%) were very similar to the rates (community sample: 42%, psychiatric disorder: 28%) in a large (n = 19,652) multinational study (MacDonald et al., 2016). These rates suggest that the prevalence of childhood trauma is underestimated, regardless of the presence of psychopathology, and that actual case-control differences in childhood trauma history probably are somewhat attenuated since MD+ status is more common in healthy controls. However, given our study's highly significant group differences in childhood trauma, the difference in minimization or denial is unlikely to explain the elevated rates found in participants with schizophrenia. Further, follow-up correlational analyses without MD+ individuals confirmed the findings.

Limitations include the use of a retrospective instrument to assess childhood trauma, which is not always in agreement with prospective reports (Baldwin et al., 2019), and which does not provide information concerning the timing of adversity. Further, as this is a cross-sectional study, we can make no inferences regarding causality. It is possible that factors preceding trauma can predispose for social cognition impairments as well as increase the risk of victimization. Another limitation is the small sample sizes, especially in within-schizophrenia follow-up analyses. A strength of the study is our ecologically valid ToM test which provides a detailed breakdown of the construct.

In conclusion, we found that physical neglect and sexual abuse in childhood were associated with reduced affective ToM in persons with schizophrenia. This highlights the importance of considering early trauma and social cognition in clinical assessments.

## CRediT authorship contribution statement

**Anja Vaskinn:** Conceptualization, Methodology, Formal analysis, Investigation, Resources, Writing - original draft, Writing - review & editing, Funding acquisition. **Ingrid Melle:** Methodology, Formal analysis, Writing - original draft, Writing - review & editing, Funding acquisition. **Monica Aas:** Methodology, Formal analysis, Writing - original draft, Writing - review & editing. **Akiah Ottesen Berg:** Conceptualization, Methodology, Formal analysis, Writing - original draft, Writing - review & editing.

## Declaration of competing interest

None.

## Appendix A Examples of MASC response categories

While the four characters prepare dinner together, one of the male characters insults, tongue-in-cheek, one of the female characters by saying that if it were up to her, they would have added five instead of two cups of cream to the sauce.

Q: What does she feel?

Response options:

A1. Insulted by his comment (correct).

A2. Hates him and wants him to leave (incorrect: overmentalizing).

A3. Amazed that he knows that she likes cream (incorrect: undermentalizing).

A4. Five cups is too much for this sauce (incorrect: no mentalizing).
